# Timeliness of routine vaccination among children and determinants associated with age-appropriate vaccination in Mongolia

**DOI:** 10.1016/j.heliyon.2020.e04898

**Published:** 2020-09-18

**Authors:** Santosh Kumar Rauniyar, Enkhtuya Munkhbat, Peter Ueda, Daisuke Yoneoka, Kenji Shibuya, Shuhei Nomura

**Affiliations:** a Department of Global Health Policy, Graduate School of Medicine, The University of Tokyo, Tokyo, Japan; b Clinical Epidemiology Division, Department of Medicine, Solna, Karolinska Institute, Stockholm, Sweden; c Department of Biosystems Science and Engineering, ETH Zurich, Basel, Switzerland; d Institute for Population Health Science, King's College London, London

**Keywords:** Vaccines, Delay, Pediatrics, Timeliness, Vaccine coverage, Vaccination, Immunization

## Abstract

**Introduction:**

Routine vaccination at the recommended age is crucial to minimize the risk of acquiring vaccine preventable diseases. This study aimed to assess the proportion of children receiving routine immunization at the recommended age and determinants of timely (age-appropriate) vaccination in Mongolia.

**Material and method:**

A total of 879 eligible children aged 12–23 months were included in this study. We investigated age-appropriate administration of Bacillus Calmette-Guerin vaccine (BCG); hepatitis B vaccine (Hep B); oral polio vaccine (OPV); pentavalent vaccine; and measles, mumps, and rubella vaccine (MMR) using Kaplan-Meier method. Multilevel logistic regression with random intercept at cluster level was used to assess the determinants of age-appropriate vaccination.

**Results:**

Overall, the crude vaccination coverage for routine vaccinations were above 90% for all vaccines except MMR1 which was 86.0% (95% CI, 83.6–88.2). While the first dose of almost all the vaccines given at birth; BCG, Hep B, and OPV0, were administered in a timely manner, a substantial proportion of second and third doses of these vaccines were not given in a timely manner with age-appropriate vaccination coverage ranging from 35.9% (32.8–39.1%) for MMR1 to 67.7% (64.5–70.7%) for OPV1 respectively. Factors associated with age-appropriate administration of the investigated vaccines included socio-economic status of household, religion of household heads, area of residence, owning mobile phone, and season of childbirth. For instance, children belonging to households from richer wealth quintile had higher possibilities of getting age-appropriate OPV1-OPV3, PE1-PE3 and MMR1 vaccines compared to those from the poorest household wealth quintile.

**Conclusion:**

Our findings suggest that the commonly used indicator ‘crude vaccination coverage’ could be supplemented by ‘age-appropriate vaccination’ to help to identify gaps in timely vaccinations and stimulate interventions in Mongolia. Factors such as household wealth quintile, place of residence and religion associated with timely vaccination in our study could be considered to promote effective intervention aiming to improve adequate vaccination coverage.

## 1 Introduction

Child vaccination is one of the simple and cost-effective public health interventions available [1, 2]. Endorsed by the World Health Assembly in 2012, the Global Vaccine Action Plan 2011–2020 calls on all countries to reach at least 90% coverage at national level [1]. In addition to high vaccination coverage, timeliness of vaccine administration, defined as administration of the vaccine at the recommended age, has received attention as an important metric to evaluate vaccination programs. An analysis of the timeliness of vaccinations showed high rates of vaccination delays across 31 low and middle-income countries (LMIC) [1, 3].

It is crucial that children are being vaccinated at the recommended age to minimize the risk of being exposed to potential life-threatening diseases [4, 5]. If children are immunized earlier than the recommended age, the immunity may be shortened [5]. For instance, measles doses given earlier than the recommended age must be repeated because of a weakened immune response [6]. Conversely, delayed vaccination increases the time between the loss of maternal antibodies and the protection from vaccine-induced immunity [4]. Delayed vaccination has been associated with increased risk of pertussis and hepatitis B [7, 8, 9].

In Mongolia, five routine vaccines, including BCG, Hep B, OPV, Pentavalent vaccine (PE: DTP, Hep B, and Haemophilus influenzae type b [Hib]), and MMR are given to children (Table 1) [10]. These vaccines are included in the National Immunization Program and given free of charge to all children throughout the country. The immunization service implemented by government of Mongolia through the public health facilities has performed well to achieve the target of at least 90% vaccination coverage at national and subnational levels throughout the country [11]. Although, the National Vaccination Program has been considered a success, with national authorities reporting crude coverages reaching up to 95% for all routine vaccines since 2011 [12], a little is known about the timeliness of the administered vaccines in the country.

**Table 1:** The routine vaccination schedule, Mongolia [15].

Name of vaccines	At birth (at 0–30 day)	2 months of age (at 61–76 day)	3 months of age (at 91–106 day)	4 months of age (at 122–137 day)	9 months of age (at 274–289 day)
BCG	BCG0				
Hep B	Hep B				
OPV	OPV0	OPV1	OPV2	OPV3	
Pentavalent (DPT, Hep B, and Hib)		Penta1	Penta2	Penta3	
MMR					MMR 1

BCG-Bacillus Calmette-Guerin vaccine; Hep B-Hepatitis B vaccine; OPV-Oral Polio vaccine; DTP-Diphtheria, Tetanus, and Pertussis vaccine; Hib-Haemophilus influenzae type b vaccine; MMR-Measles, Mumps, and Rubella vaccine; numbers indicate a dose order.

Lack of studies indicates the need for a detailed assessment of age appropriate administration of childhood vaccinations. Thus, our study aim is to evaluate timeliness of routine childhood vaccination and its coverage in Mongolia. Further, we analyzed the factors associated with age-appropriate vaccination in Mongolia. This study could serve as an important evidence to formulate effective vaccination policies in the future.

## 2 Materials and methods

### 2.1 Data source

We used data from the Mongolian Multiple Indicator Cluster Survey (MICS) conducted in 2018. MICS is a nationally representative, cross-sectional household survey program developed by the United Nations International Children's Emergency Fund (UNICEF) in 1990s, with the aim to assist countries collecting data on a wide range of health and social indicators for children and women [14]. Mongolia has implemented the MICS program since 1996 a total of eight times. The last Mongolia MICS 2018 was conducted between the period of September–December by the National Statistical Office (NSO) of Mongolia with funding support from Government of Mongolia and UNICEF.

The survey followed two-stage stratified sample design and covered all regions in Mongolia. The 2017 Population and Household registry (PHR) was used as a sampling frame. 13 strata were identified from different provinces/districts. Within each sampling stratum there was implicit stratification by urban and rural areas. 580 primary sampling units (PSUs) which were called Enumeration areas (EAs) were systematically selected with probability proportional to size. In these EAs, the clusters of 200 households were randomly selected. A total of 14,500 households were selected including 11,737 of women aged 15–49 years of age [14].

The MICS used the four sets of questionnaires: the household questionnaires, the woman's questionnaires, the man's questionnaires, and under five children's questionnaires. The questionnaire for children under five was administered to mothers (or caretakers) of the children. Through this questionnaire 6091 of children under five were selected with the response rate of 98.6%. The details of sampling methods and questionnaires were described in Mongolia MICS report [13].

### 2.2 Study population

Out of 6091 under five children, 1092 children were aged 12–23 month who were initially included in the study. From 1092 children, 213 children, who did not have mother and child health books or vaccination cards, which are official written record of vaccination history provided by Government of Mongolia [13], were excluded. In total, 879 children were included in this study.

### 2.3 Vaccination

Vaccinations assessed in this study were Bacillus Calmette-Guerin vaccine (BCG); hepatitis B vaccine (Hep B); oral polio vaccine given at birth (OPV0); oral polio, doses 1–3 (OPV1, OPV2, and OPV3); pentavalent vaccine doses 1–3, (PE1,PE2, and PE3) that include vaccines for Diphtheria, Pertussis, Tetanus, Hepatitis B and Haemophilus influenza type b, and measles, mumps, and rubella vaccine first does (MMR1) (Table 1).

### 2.4 Crude vaccine coverage and age-appropriate vaccine coverage

Crude vaccine coverage was defined as the proportion of children who received the routine vaccines regardless of the age at which the children received the vaccine.

The age-appropriate vaccination was defined as children who received a vaccination dose within the recommended age on the routine immunization schedule (Table 1) [14], plus a 15 days grace period after the due date. The grace period for age-appropriate vaccination was decided based on previous studies [4, 6]. The age at which the vaccine given was calculated by subtracting the date of birth from date of the vaccination. Children receiving the vaccination after the recommended age-range were considered to have received delayed vaccination. Early vaccination was defined as vaccination given before the recommended age-range. Children who had been marked as not given vaccination or marked as given vaccination, but no date found on the mother and child health book or vaccination card were considered as not having received the vaccination.

### 2.5 Statistical analysis

The crude and age-appropriate vaccine coverage with 95% confidence interval (CI) were calculated for each vaccine dose. Kaplan-Meier product limit method was employed to analyze each dose of vaccines received by children within the given immunization schedule provided by National Immunization program (NIP), Mongolia. To take into account for the survey design which is multi-stage sampling method, all the analyses were adjusted with sampling weight.

Next, we used multilevel logistic regression, with random intercept at PSU level considering the survey design, to investigate the association between the age-appropriate vaccination and socioeconomic variables, as well as characteristics of the children and their parents, including gender of the children, mothers' age, mothers' education, socio-economic status of households, religion of household heads, ethnicity, area of residence, mothers’ occupation, and season of child birth. The backward stepwise variable selection method with cut-off level at p < 0.05 of each coefficient was used to select covariates. The regression models included random effects at cluster level to control for correlation between cluster and region. Each random effect was assumed to follow the multivariate normal distribution. The restricted maximum likelihood method was used to estimate the regression parameters. STATA/IC 14 was used for the data analysis, and p < 0.05 was considered statistically significant.

We used the STROBE cross-sectional reporting guidelines, the standard guidelines to report cross-sectional study [35].

### 2.6 Ethics approval

Ethical approval was not needed for this study which used publicly available data from MICS.

## 3 Results

### 3.1 Sample characteristics

There were 879 eligible children included in the analysis: 49.4% (n = 434) were male, 55.8% (490) had mothers aged between 25 and 35 years and 36.6% (322) had mothers with secondary school or vocational education background (Table 2). Out of total sample population, 45.6% (400) were Buddhist, 78.5% (690) were of Khalkh ethnicity and 20.2 (177) lived in rural areas (Table 2). The details of vaccination included in our study is provided in Table 1.

**Table 2:** Sample characteristic of 890 children aged 12–23 months, Mongolia, 2013.

Variables	Number	Proportion (%)
Gender		
Male	434	49.4
Female	445	50.6
Mother's age		
16–24	147	16.7
25–35	490	55.8
36–49	214	24.4
Missing	28	3.1
Mother's education		
None	33	3.8
Primary of basic level	137	15.6
Secondary school or vocational	322	36.6
College or university	387	44.0
Socioeconomic status∗		
Poorest	169	19.2
Poorer	177	20.1
Middle	234	26.6
Richer	127	14.5
Richest	172	19.6
Religion		
No religion	407	46.4
Buddhist	400	45.6
Muslim	33	3.7
Other	39	4.3
Ethnicity		
Khalkh	690	78.5
Kazakh	33	3.8
Other	154	17.5
Missing	2	0.2
Area of residence		
Capital city/Ulaanbaatar	434	49.3
Aimag center	169	19.2
Soum center	99	11.3
Rural	177	20.2
Season of childbirth		
Winter	233	26.5
Spring	239	27.2
Summer	228	25.9
Autumn	179	20.4

∗ Socio-economic status of household was defined using wealth index based on information on the ownership of consumer goods, dwelling characteristics, water and sanitation, and other characteristics that are related to the household’s wealth. Random effects at PSU level to account for survey design [13].

### 3.2 Crude and age-appropriate vaccine coverage

The crude vaccine coverage ranged from 86.0% (95% CI, 83.6–88.2%) for MMR1 to 98.2% (97.1–98.7%) for Hep B and OPV0. The vaccines which are given right after birth at maternal hospitals (e.g. BCG, Hep B, and OPV0) had higher age-appropriate vaccination coverages than other vaccines (Table 3A).

**Table 3:** Crude and age-appropriate vaccination coverage in Mongolia (n = 879).

Vaccine doses	Crude coverage		Age-appropriate coverage		Early vaccination	Delayed vaccination
	Number	Proportion (95% CI)	Number	Proportion (95% CI)	Proportion, 95% CI	Proportion, 95% CI
BCG	859	97.7 (96.5–98.5)	806	91.7 (89.7–93.4)	-	-
Hep B	863	98.2 (97.1–98.9)	804	91.6 (89.5–93.2)	-	-
OPV0	863	98.2 (97.1–98.9)	805	91.7 (89.6–93.3)	-	-
OPV1	848	96.5 (95.0–97.5)	594	67.7 (64.5–70.7)	13.1 (11.0–15.5)	19.2 (16.8–22.0)
OPV2	824	93.8 (92.0–95.2)	513	58.4 (55.1–61.6)	13.3 (11.2–15.7)	28.3 (25.4–31.4)
OPV3	800	91.1 (89.0–92.8)	410	46.7 (43.4–50.0)	12.6 (10.5–14.9)	40.8 (37.6–44.1)
Penta1	838	95.4 (93.8–96.6)	573	65.3 (62.1–68.4)	10.9 (9.0–13.1)	23.8 (21.1–26.8)
Penta2	820	93.4 (91.5–94.8)	518	59.0 (55.7–62.2)	7.7 (6.1–9.7)	33.3 (30.2–36.5)
Penta3	793	90.3 (88.2–92.1)	424	48.2 (44.9–51.5)	8.6 (6.9–10.6)	43.2 (40.0–46.5)
MMR1	756	86.0 (83.6–88.2)	315	35.9 (32.8–39.1)	19.4 (16.9–22.2)	44.7 (41.4–48.0)

BCG-Bacillus Calmette-Guerin vaccine; Hep B-Hepatitis B vaccine; OPV-Oral Polio vaccine(doses 0–3); PE- DTP-Diphtheria, Tetanus, and Pertussis vaccine; Hib-Haemophilus influenzae type b vaccines (doses 1–3); MMR1-Measles, Mumps, and Rubella vaccine; numbers indicate a dose order; CI-Confidence interval.

Random effects at PSU level to account for survey design.

**Figure 1:**
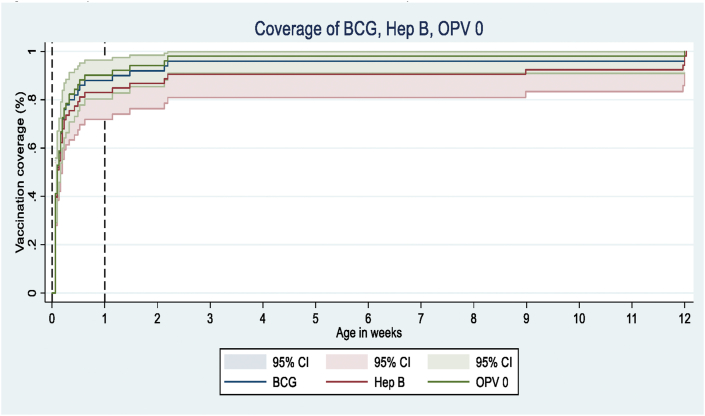
Proportion of children immunized with the BCG, Hep B, and OPV0. BCG-Bacillus Calmette-Guerin vaccine, Hep B-Hepatitis B vaccine, OPV0-Oral Polio vaccine at birth; for the sake of clarity, x axis is truncated at 6 months of age.

Figure 1 shows the age-appropriate coverage of BCG, Hep B, and OPV0 vaccines received by children over time, estimated using the Kaplan-Meier product limit method. As shown in Table 3A, 98.2 (95% CI, 97.1–98.9%) of the children were vaccinated for Hep B and OPV0, within the recommended age-range, while 97.7% (96.5–98.5%) were age-appropriately vaccinated for BCG. Median (inter quartile range [IQR]) delay among those with a delayed vaccination was 55 (28–365) days for BCG, 147 (30–365) days for Hep B, and 75 (30–365) days for OPV0.

**Figure 2:**
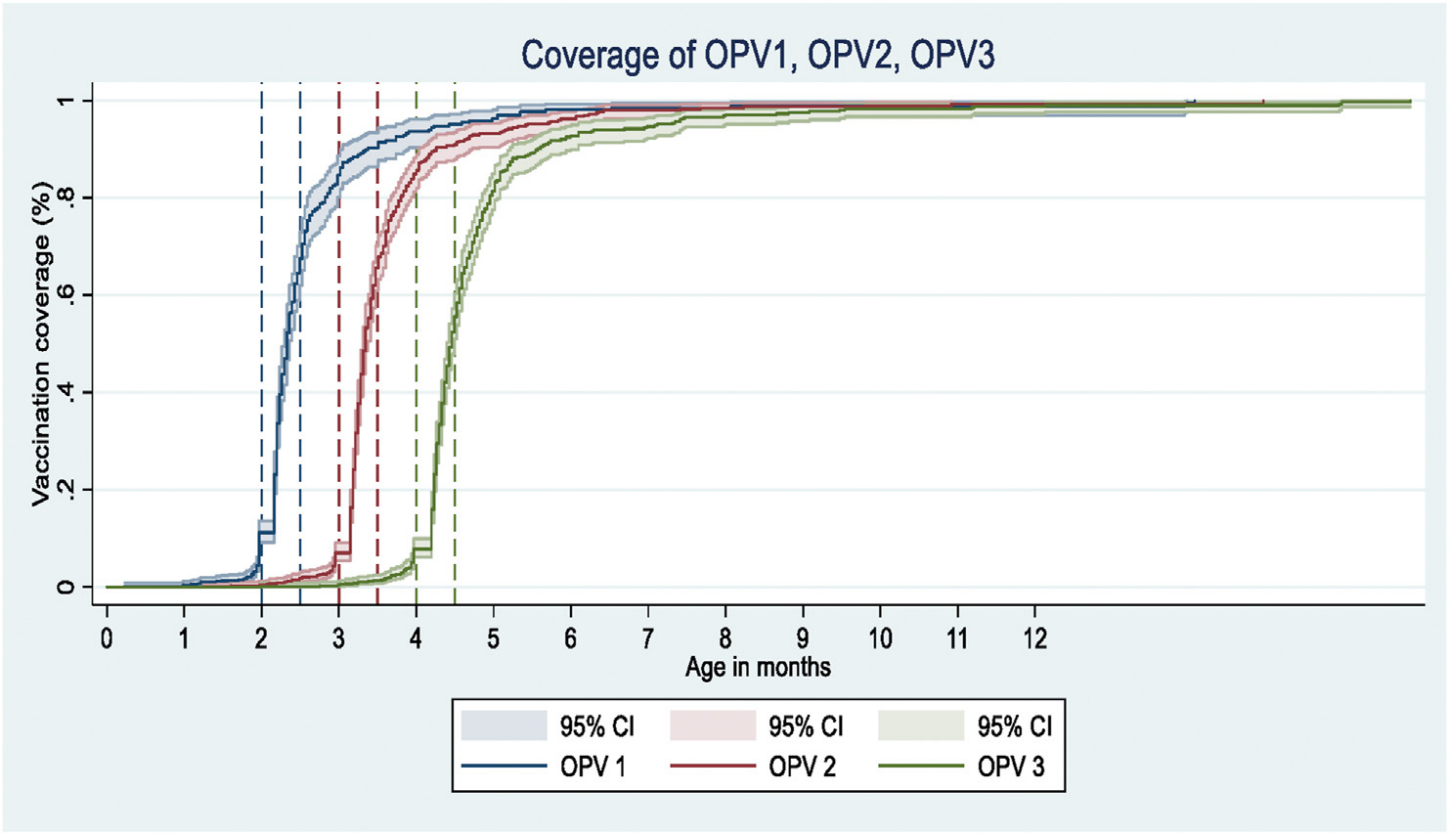
Proportion of children immunized with the OPV1-3. OPV1-first dose of Oral Polio vaccine; OPV2-second dose of Oral Polio vaccine; OPV3-third dose of Oral Polio vaccine; x axis is truncated at 10 months of age.

Figure 2 shows the age-appropriate coverage of OPV1-OPV3 (OPV1, OPV2, and OPV3) vaccines received by children over time. As shown in Table 3A, for OPV1, 67.7% (95% CI, 64.5–70.7%) of the children received age-appropriate vaccinations; for OPV2 and OPV3, these number were 58.4% (55.1–61.6%) and 46.7% (43.4–50.0%), respectively. The proportions of earlier vaccination for OPV1, OPV2, and OPV3 were 13.1% (11.0–15.5%), 13.3% (11.2–15.5%), and 12.6% (10.5–14.9%), respectively. The proportions of delayed vaccination for OPV1, OPV2, and OPV3 were 19.2% (16.8–22.0%), 28.3% (25.4–31.4%), and 40.8% (37.6–44.1%), respectively(Table 3B). The median interquartile range (IQR) delays among those with a delayed vaccination for OPV1, OPV2, and OPV3 were 63 (62–63) days, 110 (108–113) days, and 145 (142–149) days, respectively.

**Figure 3:**
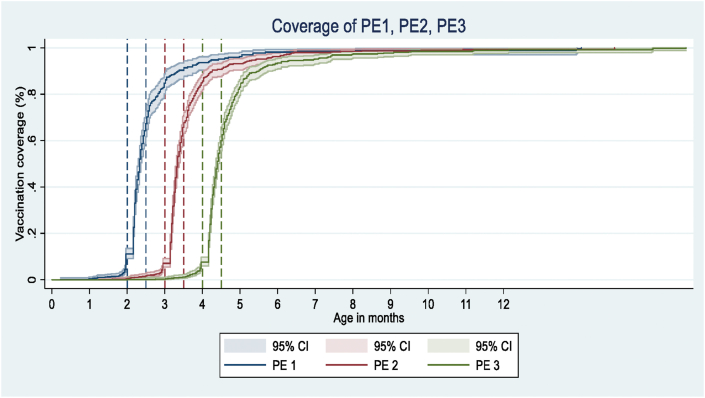
Proportion of children immunized with the PE1-3. PE1-first dose of Pentavalent vaccine; PE2-second dose of Pentavalent vaccine; PE3-third dose of Pentavalent vaccines; x axis is truncated at 10 months of age.

Figure 3 presents children who received age-appropriate PE1-PE3 (PE1, PE2, and PE3) vaccines over time. For PE1-PE3 vaccines, 65.3% (95% CI, 62.1–68.4%), 59.0% (55.7–62.2%), and 48.2% (44.59–51.5%) children were vaccinated within the recommended age-range respectively (Table 3A). The proportions of earlier vaccination for PE1, PE2, and PE3 were 10.9% (9.0–13.1%), 7.7% (6.1–9.7%), and 8.6% (6.9–10.6%), respectively. The proportion of delayed vaccination for PE1, PE2, and PE3 were 23.8% (21.1–26.8%), 33.3% (30.2–36.5%), 43.2% (40.4–46.5%), respectively (Table 3B). Median (IQR) delays among those with a delayed vaccination for PE1, PE2, and PE3 were 63 (63–64) days, 113 (111–115) days, and 147 (143–151) days, respectively.

**Figure 4:**
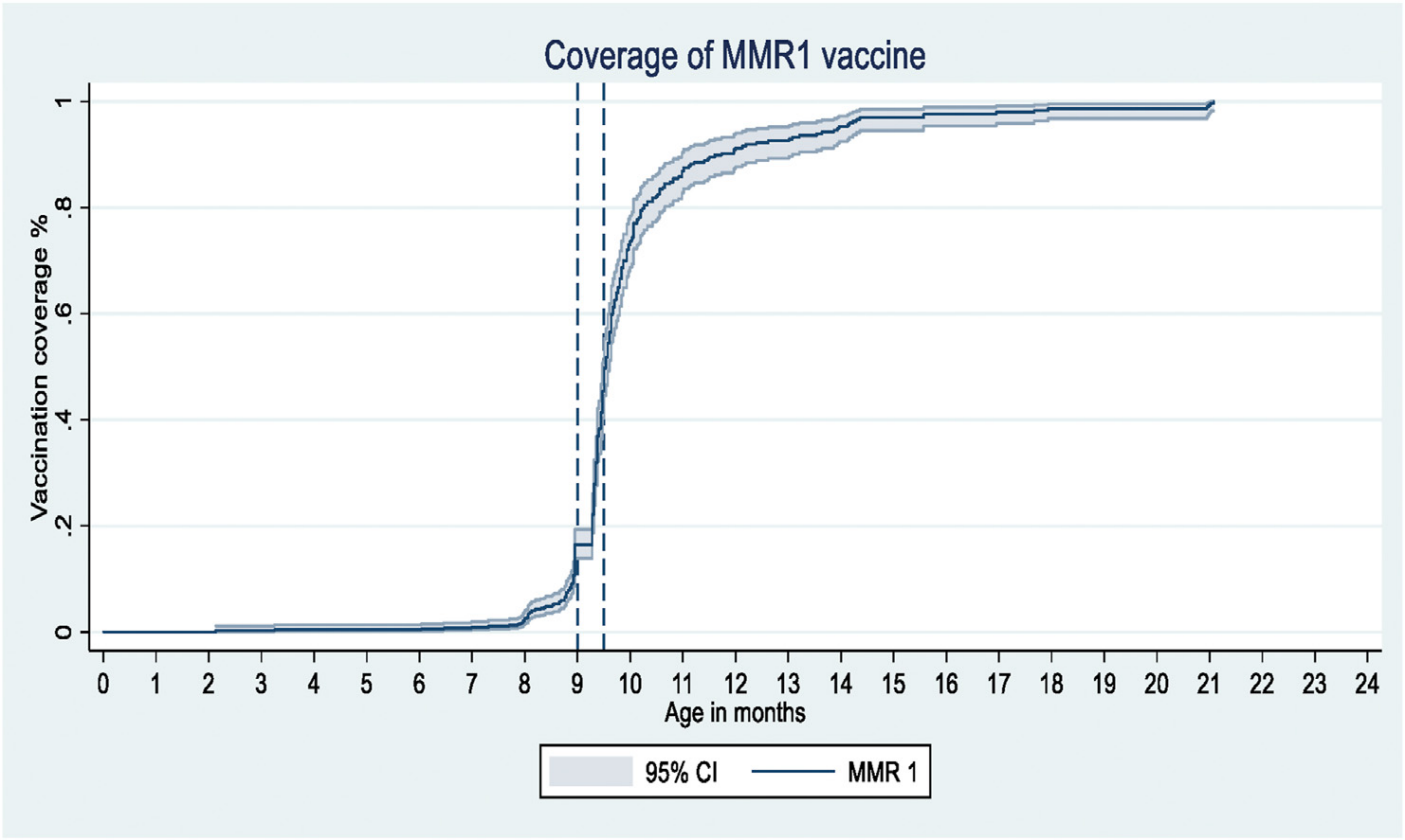
Proportion of children immunized with the MMR. MMR-Measles, Mumps, and Rubella vaccine; x axis is truncated at 14 months of age.

Figure 4 presents children who received age-appropriate MMR1 over time. 35.9% (95% CI, 32.8–39.1%) of the children were given the vaccination within the recommended age-range (Table 3A). The proportions of earlier vaccination and delayed vaccination were 19.4% (16.9–22.2%) and 44.7% (41.4–48.0%), respectively (Table 3B). Median (IQR) delay among those with a delayed vaccination was 291 (273–294) days (Table 3).

**Figure 5:**
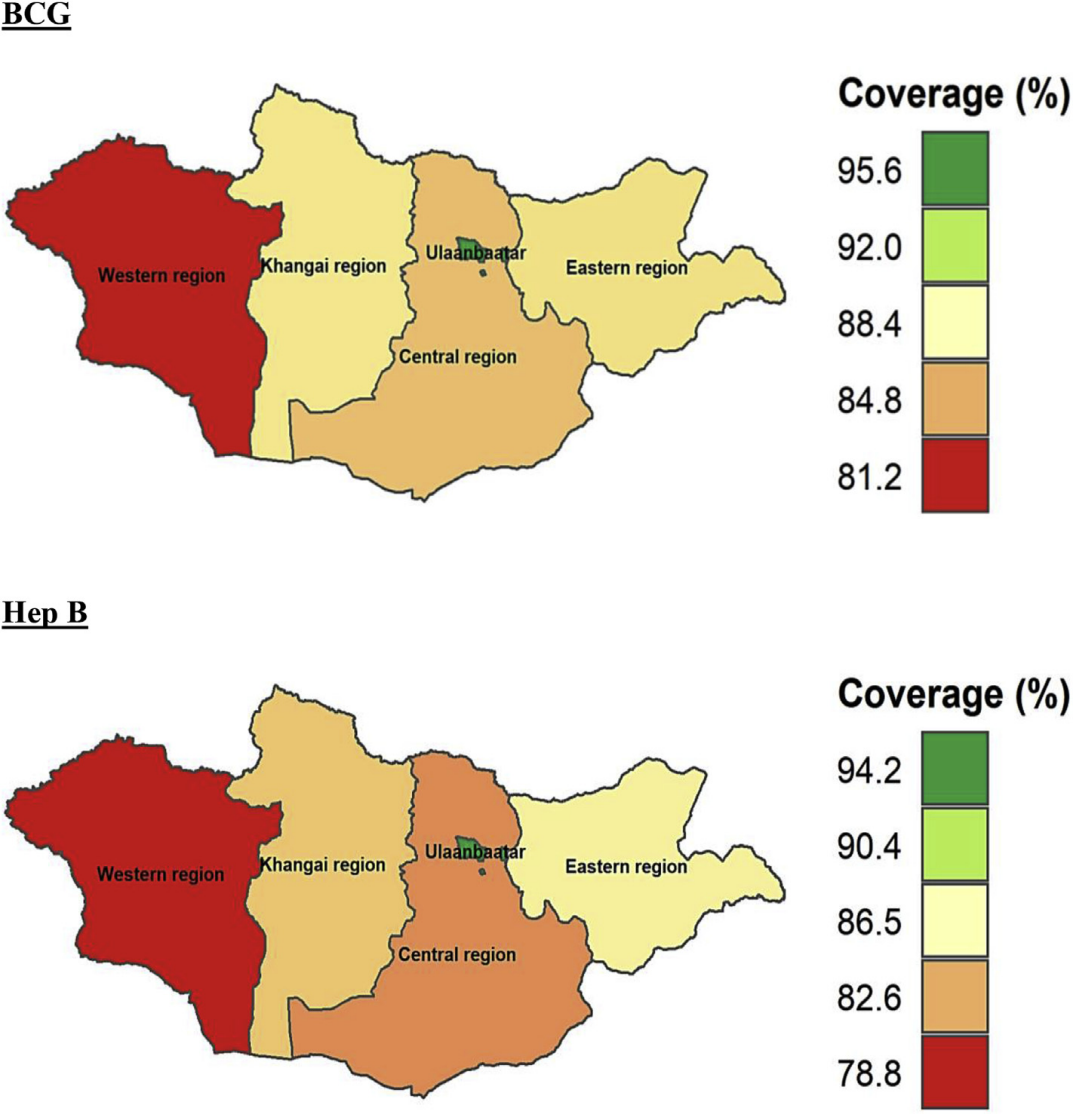
Age-appropriate coverage of BGC and Hep B vaccines at subnational levels in Mongolia. BCG – Bacillus Calmette-Guerin vaccine; Hep B – Hepatitis B vaccine; number indicates the dose. ∗ All the choropleth maps were generated using R programming software.

**Figure 6:**
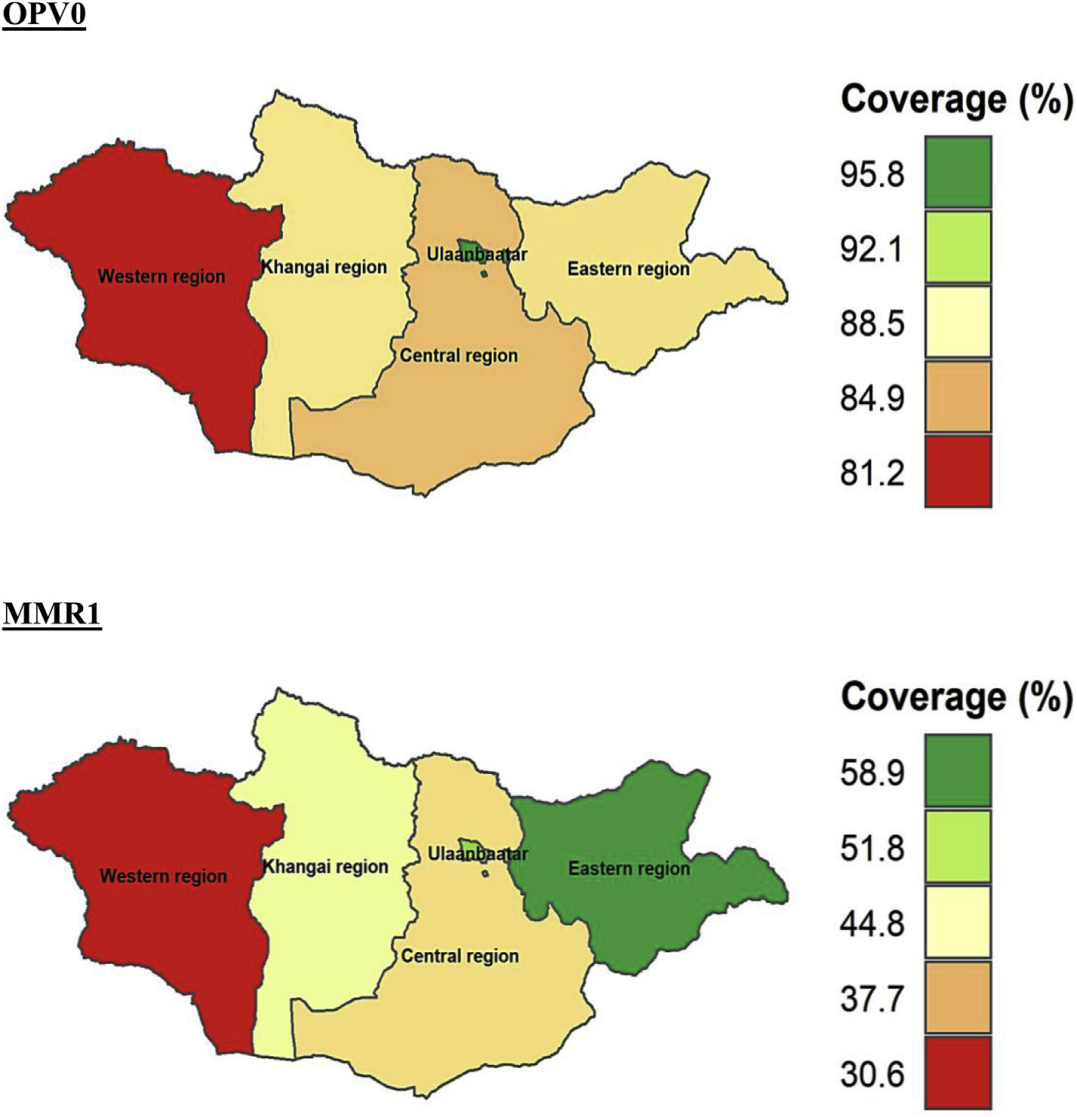
Age-appropriate coverage of OPV0 and MMR1 vaccines at subnational levels in Mongolia. OPV –Oral polio vaccine; MMR – Measles, mumps and rubella vaccine; number indicates the dose. ∗ All the choropleth maps were generated using R programming software.

**Figure 7:**
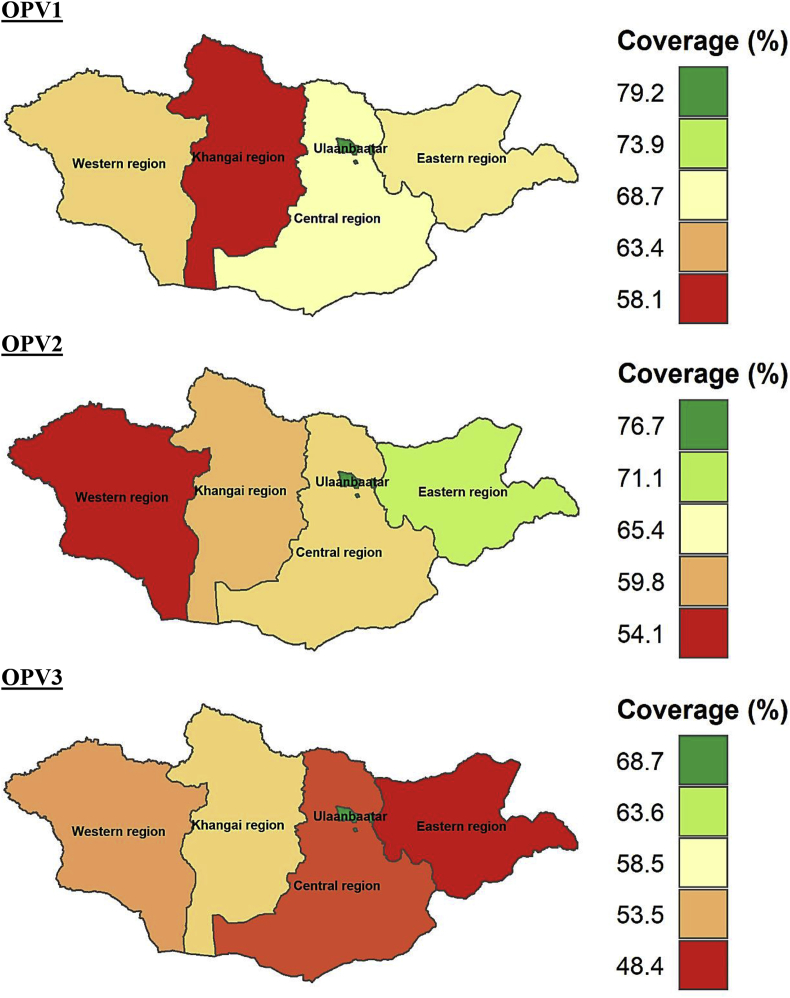
Age-appropriate coverage of OPV1-OPV3 vaccines at subnational levels in Mongolia. OPV – Oral polio vaccine; number indicates the dose. ∗ All the choropleth maps were generated using R programming.software.

**Figure 8:**
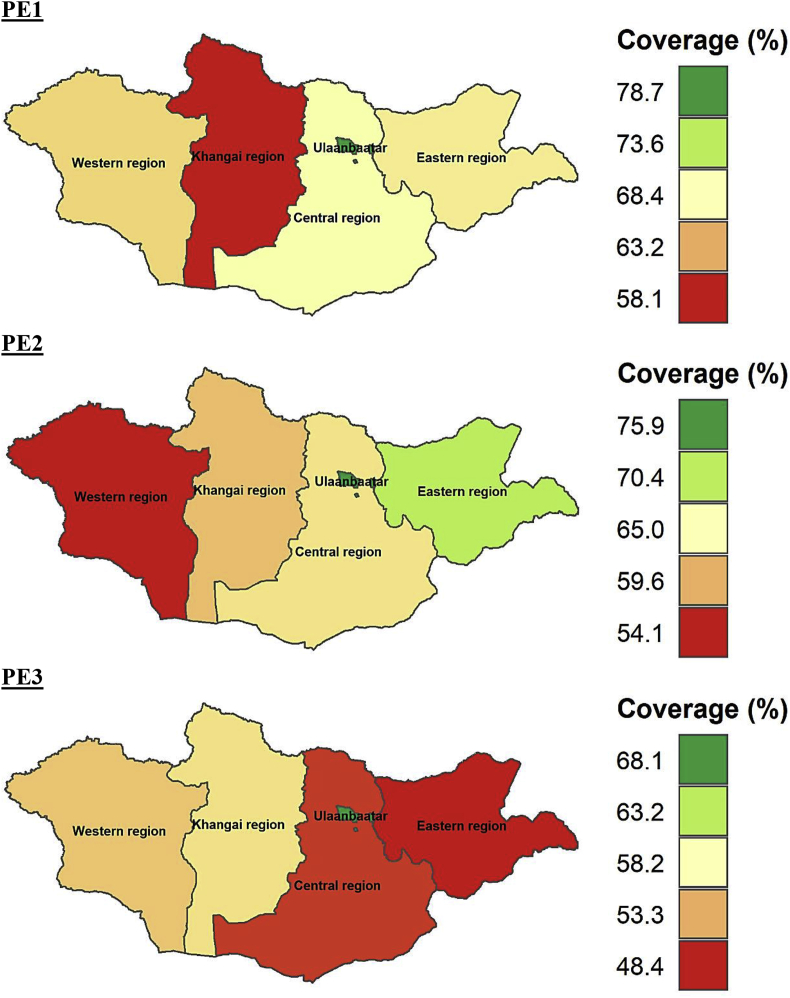
Age-appropriate coverage of PE1-PE3 vaccines at subnational levels in Mongolia. PE –pentavalent vaccine; Pertussis, tetanus, Hepatitis B and Haemophilus influenza type b; number indicates the dose. ∗ All the choropleth maps were generated using R programming software.

At subnational levels, the age-appropriate coverage of all the vaccines studied differed significantly. The coverage of all the vaccines (BCG, Hep B, OPV0, OPV1-OPV3, PE1-PE3, and MMR1) was higher in Ulaanbaatar which is the capital city of Mongolia compared to other regions. The timely coverage of BCG, Hep B, OPV0, MMR1, OPV2, and PE2 was lowest in Western region compared to other parts of Mongolia. The age-appropriate coverage of BCG, Hep B, OPV0, OPV1-OPV3, PE1-PE3, and MMR1 at subnational level is provided in Figures 5, 6, 7, and 8.

### 3.3 Factors associated with age-appropriate vaccination of BCG, Hep B and OPV0

The significant results from multilevel logistic regression models is shown in Table 4. The regression analyses showed that the children in Buddhist households had significantly higher odds of receiving age-appropriate BCG, Hep B and OPV0 vaccines (odds ratio [OR], 2.12, 95% CI, 1.17–3.84) for BCG, (1.74, 1.06–2.85) for Hep B, and (2.07, 1.14–3.78) for OPV0 respectively) than those having no religion. Compared to the children from rural area, those living in urban areas had higher odds of receiving age-appropriate vaccination (OR, 3.71 (2.01–6.84 for BCG), 3.56 (2.13–5.94) for Hep B, and 3.99 (2.13–7.46) for OPV0 respectively). Season of childbirth was also significant with timely vaccination of BCG, Hep B and OPV0 vaccines. Children born in summer had significantly higher odds of receiving age-appropriate vaccination (OR, 2.64 (1.22–5.70) for BCG, 2.13 (1.08–4.23) for Hep B), and 2.68 (1.22–5.87) for OPV0 respectively (Table 4).

**Table 4:** Multilevel logistic regression results for BCG, Hep B, and OPV0.

Variables	Odds ratio (95% confidence interval) for age appropriate vaccination		
	BCG	Hep B	OPV0
Religion			
No religion	1.00 (ref)	1.00 (ref)	1.00 (ref)
Buddhist	2.12 (1.17–3.84)∗	1.74 (1.06–2.85)∗	2.07 (1.14–3.78)∗
Muslim	18.17 (0.32–101.75)	5.57 (0.20–153.90)	19.24 (0.33–113.66)
Place of residence			
Rural	1.00 (ref)	1.00 (ref)	1.00 (ref)
Urban	3.71 (2.01–6.84)∗∗∗	3.56 (2.13–5.94)∗∗∗	3.99 (2.13–7.46)∗∗∗
Season of childbirth			
Winter	1.00 (ref)	1.00 (ref)	1.00 (ref)
Spring	1.98 (1.00–3.92)	2.22 (1.22–4.05)∗∗	2.21 (1.11–4.40)∗
Summer	2.64 (1.22–5.70)∗	2.13 (1.08–4.23)∗	2.68 (1.22–5.87)∗
Autumn	1.85 (0.90–3.80)	1.81 (0.97–3.39)	1.86 (0.90–3.85)

BCG-Bacillus Calmette-Guerin vaccine; Hep B-Hepatitis B vaccine; OPV0-Oral Polio vaccine at birth; ref-reference; ∗p < 0.05; ∗∗p < 0.01; ∗∗∗p < 0.001.

Random effects at PSU level to account for survey design.

### 3.4 Factors associated with age-appropriate vaccination of OPV1-OPV3, PE1-PE3, and MMR1

The result of regression analyses for OPV1-OPV3, PE1-PE3, and MMR1 is provided in Tables 5 and 6. For age-appropriate vaccination of OPV1, PE1 and, PE2 vaccines, households’ religion was significantly associated with age-appropriate administration of OPV and PE vaccine doses. Particularly, children born in the Muslim religion households had significantly lower possibilities of receiving age-appropriate OPV1,PE1, and PE2, vaccines compared to children in households following no religion. Children born in richer and richest households were approximately 2 times or even higher possibilities of receiving age-appropriate OPV1-OPV3 and PE1-PE3 vaccines compared to those born in poorest households as shown in Table 5. Children born in winter were significantly less likely to receive timely vaccination for all the does of OPV and PE vaccines compared to the children born in summer or spring(Table 5). However, the multilevel regression model did not show significant association of timely administration of MMR1 vaccine with socioeconomic status and season of childbirth. It was important to note that the children with the parents owning mobile phone had significantly higher odds of timely administration of first and second doses of OPV and PE vaccines (odds ration OR, 3.32 (1.24–8.91) for OPV1, 2.77 (1.17–6.52) for OPV2, 3.27 (1.22–8.73) for PE1, and 2.78 (1.18–6.56) for PE2 respectively). Children born in urban area were approximately 2 times more likely to receive age-appropriate doses of OPV1-OPV3, PE1_PE3 and MMR1 vaccines compared to those born in rural area as shown in Table 5 and Table 6.

**Table 5:** Multilevel logistic regression results for OPV1-OPV3 and PE1-PE3.

Variables	Odds ratio (95% confidence interval) for age appropriate vaccination					
	OPV1	OPV2	OPV3	PE1	PE2	PE3
Religion						
No religion	1.00 (ref)	1.00 (ref)	1.00 (ref)	1.00 (ref)	1.00 (ref)	1.00 (ref)
Buddhist	0.97 (0.68–1.38)	0.87 (0.61–1.25)	1.04 (0.76–1.45)	0.97 (0.68–1.39)	0.90 (0.63–1.30)	1.03 (0.75–1.43)
Muslim	0.14 (0.03–0.73)∗	0.20 (0.04–0.92)	0.34 (0.09–1.38)	0.14 (0.03–0.72)∗	0.20 (0.05–0.90)∗	0.35 (0.09–1.39)
Wealth quintile						
Poorest	1.00 (ref)	1.00 (ref)	1.00 (ref)	1.00 (ref)	1.00 (ref)	1.00 (ref)
Poorer	1.64 (0.89–3.03)	1.81 (1.00–3.30)∗	1.44 (0.81–2.55)	1.61 (0.87–2.97)	1.96 (1.07–3.58)∗	1.37 (0.78–2.41)
Middle	2.49 (1.33–4.64)∗	3.24 (1.73–6.07)∗∗∗	2.15 (1.20–3.85)∗	2.54 (1.36–4.74)∗∗	3.43 (1.83–6.40)∗∗∗	2.06 (1.16–3.65)∗
Richer	2.79 (1.40–5.57)∗	2.78 (1.43–5.41)∗∗	1.84 (1.02–3.32)∗	2.70 (1.35–5.40)∗∗	2.86 (1.47–5.58)∗∗	1.66 (0.92–2.98)
Richest	2.35 (1.15–4.80)∗	4.32 (2.12–8.80)∗∗∗	2.34 (1.25–4.40)∗∗	2.42 (1.19–4.94)∗	4.27 (2.09–8.71)∗∗∗	2.18 (1.17–4.06)∗
Owns mobile phone						
No	1.00 (ref)	1.00 (ref)	1.00 (ref)	1.00 (ref)	1.00 (ref)	1.00 (ref)
Yes	3.32 (1.24–8.91)∗	2.77 (1.17–6.52)∗	1.49 (0.63–3.52)	3.27 (1.22–8.73)∗	2.78 (1.18–6.56)∗	1.50 (0.64–3.51)
Place of residence						
Rural	1.00 (ref)	1.00 (ref)	1.00 (ref)	1.00 (ref)	1.00 (ref)	1.00 (ref)
Urban	1.88 (1.15–3.09)∗	1.78 (1.07–2.94)∗	1.90 (1.21–2.96)∗∗	1.83 (1.12–3.00)∗	1.76 (1.06–2.91)∗	1.85 (1.19–2.88)∗∗
Season of childbirth						
Winter	1.00 (ref)	1.00 (ref)	1.00 (ref)	1.00 (ref)	1.00 (ref)	1.00 (ref)
Spring	3.01 (1.85–4.91)∗∗∗	1.71 (1.03–2.83)∗	1.76 (1.13–2.74)∗	2.94 (1.81–4.78)∗∗∗	1.62 (0.97–2.68)	1.75 (1.13–2.72)∗
Summer	4.50 (2.53–7.99)∗∗∗	2.40 (1.42–4.07)∗∗	2.18 (1.32–3.62)∗∗	4.45 (2.51–7.88)∗∗∗	2.14 (1.26–3.63)∗∗	1.99 (1.21–3.28)∗∗
Autumn	2.76 (1.62–4.68)∗∗∗	1.44 (0.87–2.38)	1.25 (0.79–1.97)	2.64 (1.56–4.46)∗∗∗	1.40 (0.84–2.32)	1.19 (0.76–1.88)

OPV1-first dose of Oral Polio vaccine; OPV2-second dose of Oral Polio vaccine; OPV3-third dose of Oral Polio vaccine; PE1-first dose of Pentavalent vaccine; PE2-second dose of Pentavalent vaccine; PE3-third dose of Pentavalent vaccine; ref-reference; ∗p < 0.05; ∗∗p < 0.01; ∗∗∗p < 0.00.

Random effects at PSU level to account for survey design.

**Table 6:** Multilevel logistic regression results for MMR1.

Variables	Odds ratio (95% confidence interval)
Religion	
Muslim	1.00 (ref)
No religion	1.12 (0.80–1.57)
Buddhist	0.30 (0.04–2.34)
Wealth Quintile	
Poorest	1.00 (ref)
Poorer	1.30 (0.70–2.41)
Middle	1.13 (0.61–2.08)
Richer	1.08 (0.55–2.12)
Richest	1.64 (0.81–3.29)
Owns mobile phone	
No	1.00 (ref)
Yes	1.06 (0.38–2.93)
Place of residence	
Rural	1.00 (ref)
Urban	2.02 (1.20–3.40)∗∗
Season of childbirth	
Winter	1.00 (ref)
Spring	0.85 (0.51–1.42)
Summer	0.94 (0.57–1.56)
Autumn	0.69 (0.42–1.12)

MMR-Measles, Mumps, and Rubella vaccine; ref-reference; ∗∗p < 0.01.

Random effects at PSU level to account for survey design.

## 4 Discussion

We evaluated the age-appropriate coverage of routine vaccines in children aged 12–23 month in Mongolia. The study findings suggest that untimely vaccination in Mongolia is common and the assessment of timeliness of vaccination is important to evaluate the effectiveness of vaccination and its coverage. We found that the highest age-appropriate vaccination coverage had been attained for BCG, Hep B, and OPV0. A possible explanation for this could be that those vaccines are given at birth to children in maternity hospitals or wards. Mongolia has high rate of hospital-based delivery; in 2017, this was around 99.6% [15].

Lower age-appropriate vaccination coverage was observed for later doses for OPV (dose 1–3), PE1-PE3 and MMR1 vaccines. According to the routine vaccination schedule, all the doses of OPV and PE vaccines are given to a child at the same time. Previous studies in other countries have suggested that vaccine delays and parental hesitancy or avoidance towards vaccination might be related to fears concerning simultaneous vaccination at a single visit [16]. Moreover, other studies mentioned that side effects like fever, swelling, and pain at injection site after the first vaccinations could lead children to be less likely to receive the subsequent doses [17, 18].

A substantial proportion of children, who received MMR1 vaccination, were not administered within the recommended age-range. The age-appropriate coverage for MMR in this study was 35.9% which is substantially lower than median timely MMR1 coverage that is 51.0% in other LMIC assessed in 2010 [19]. About 93–95% coverage is considered to build measles's herd immunity in a community [4]. Mongolia experienced a big measles outbreak affecting more than 50,000 people in 2015–2016 which occurred shortly after the country was certified measles-free by WHO in 2014 [20]. Thus, efforts to reduce the number of susceptible individuals through timely vaccinations could be important to avoid a recurrent outbreak.

In this study we also identified factors associated with the age-appropriate vaccine administration. We found that household wealth quintile, place of residence, religion, and season of childbirth were significantly associated with age-appropriate vaccination.

Higher socio-economic status of the household was significantly associated with timely vaccination in this study. Children from richer and richest households were approximately three times more likely to receive age-appropriate OPV1-OPV3 and PE1-PE3 vaccines as compared to children living in poorest households. In accordance with these findings, many studies in other settings have reported that lower socio-economic status is a risk factor for delayed vaccination or non-vaccination; this association has partly been attributed to the cost of reaching the health facility [4, 21, 22, 23]. Although Mongolia offers free routine vaccination service for all children, transportation costs are not covered. Therefore, outreach services to vaccinate socioeconomically disadvantaged children may be considered in National Immunization Plan. A previous study on the vaccine hesitancy in Mongolia noted that younger and poorer parents were less likely to have their children vaccinated on time. Reasons included were costs of transportation, fears of vaccines, insufficient information on vaccine safety, lack of communication with healthcare workers, and uncomfortable experiences of health facilities [24, 29].

In this study, we found that owning mobile phone and place of residence were significantly associated with age-appropriate vaccination. Children born in urban area were approximately two times more likely to get age-appropriate vaccination. This result has been consistent with the results from previous studies conducted in Ghana and Nigeria [25, 26]. It was important to note that children whose parents owned mobile phones had significantly higher possibilities of having timely coverage of first and second doses of OPV and PE vaccines. This could be because of constant reminder message sent on mobile phone regarding vaccination dates and its administration. Several studies have shown that mobile health (mHealth) intervention improves the vaccination coverage [27, 28]. Compared to the children born in families without religion, those belonging to Buddhist families had significantly higher odds of getting age appropriate vaccination for BCG Hep B and OPV0. Similar outcome was observed in one of the studies conducted in India [29]. Some previous studies suggest that religion and caste can influence the parental beliefs and attitude towards health-seeking behaviors, including vaccination decision [30, 31]. However, considering these results, extensive qualitative studies focused on parental attitude towards vaccination among different religious groups would provide better insight.

Season of childbirth was significantly associated with age-appropriate coverage for first dose of OPV and PE vaccines. Children born in summer and spring were twice likely to be vaccinated within the recommended age-range. These findings are in line with those from previous studies on timely vaccination in Norway and Bangladesh [5, 32, 33]. Although speculative, the reason of the increased risk of untimely vaccination among children born in winter and autumn has been suggested to be related to seasonal flu [34].

The findings of this study have implications for research and public health measures aimed at improving vaccination coverage in Mongolia. We showed that, although high crude vaccination rates have been achieved in Mongolia, a substantial proportion of the administered vaccines were not provided within the recommended age-range. The coverage of age-appropriate vaccination should be considered in the assessment of vaccination coverage in Mongolia. Our findings of factors associated with age-appropriate vaccinations may also be considered in the design of interventions aiming at improving coverage of age-appropriate vaccinations.

### 4.1 Limitations

This study has several limitations. First, only children who had vaccination records in the mother and child health book (the vaccination card) were included. Exclusion of children without vaccination records might lead to overestimation of the vaccination coverage and timeliness if these children were less likely to receive adequate vaccinations. Children who were excluded from our analyses due to missing data on vaccination were more likely to be from the poorest household as compared with those included in the study. Second, age-appropriate vaccination coverage among children can be influenced by many other factors, including those related to access to health care services, knowledge, attitudes, and practices of parents and providers. The variables investigated in this study were limited to those available in MICS. Third, because of a cross-sectional sample of children aged 12–23 months, the timing of assessment of vaccine coverage differed depending on the age of child at which the survey was conducted. This meant that the crude vaccination rates could not be properly assessed as some children may have received a delayed vaccination after survey participation. Finally, both early and delayed vaccinations were analyzed as a single category. Investigation of each of these types of untimely vaccinations is a topic for future studies.

## Declarations

### Author contribution statement

S. Rauniyar: Conceived and designed the experiments; Performed the experiments; Wrote the paper.

E. Mankhbat: Performed the experiments.

P. Ueda: Analyzed and interpreted the data; Contributed reagents, materials, analysis tools or data.

S. Nomura: Analyzed and interpreted the data.

D. Yoneoka and K. Shibuya: Contributed reagents, materials, analysis tools or data.

### Funding statement

This research did not receive any specific grant from funding agencies in the public, commercial, or not-for-profit sectors.

### Competing interest statement

The authors declare no conflict of interest.

### Additional information

No additional information is available for this paper.
